# The Shortage of M.A.B. Nurses

**Published:** 1918-10-19

**Authors:** 


*To the Editor of* The Hospital.

### The Shortage of M.A.B. Nurses.

*To the Editor of* The Hospital.

Sir,—To create dissatisfaction among the M.A.B. staff is far from my mind, and I do not wish anything published that may lead to it, but as a nurse who has worked in London's largest general hospital, and for two years in a military hospital, besides three of the M.A.B. institutions, I think much might be done to relieve the present shortage. In the first place, why should infectious nurses be looked on as doing lower-class work than other hospitals, either military or general? What can be more heroic than fighting daily unseen germs? Disease thus contracted in many cases means the sacrifice of all future hopes because the victim will never again be strong enough to nurse. It is true the M.A.B. always tries to find occupation for their weakened staff, but, were the same thing to happen to a nurse in the Forces, she would be honourably discharged with a badge and an adequate pension. Not so with us; our lot is just to creep along the side-path left as best we can. Even the medical superintendents do not put outside nurses on the same level as their own. For instance, we have a nurse brought in from another hospital. She is treated in a separate room or isolation ward, where she is undisturbed by the children. Our own nurses have to suffer the continual disturbance and racket of being warded in the big wards on the same level as the patients, and this even if they are not suffering from an infectious illness; and in nine cases out of ten the outside nurse has not fallen a victim in the course of her work.

Then, too, what reason is there for not granting privileges, such as reduced railway fare, to us as well as them? We are told that our work is of national importance, and so it is. What is of more value to the nation than the present-day children? And why is the nursing of discharged soldiers any different from that of those whose day's work is not yet over? The same amount of time is now given to training—*i.e.*, three years. The only difference is that general-trained nurses get the greatest consideration and highest pay when finished. Although I am certain general training does not turn out better nurses, it is true you go over a wider ground, but there is not the time to do the subjects thoroughly. I am convinced that in no other class of hospital are patients given such individual care and aseptic treatment as in the modern M.A.B. institutions.

Another very important point. Although the country is urging economy on all other hospitals, the hospitals have not hesitated to supply all their various branches with a smart uniform, and well laundered. The M.A.B. uniform and laundry is, well—the limit. I never feel I want to face anyone while wearing it. It may be said that in pre-war days the starting salaries were higher, but the off-duty time is necessarily arranged differently. Other nurses have as a rule only one day per month off duty on which to provide food. M.A.B. nurses have six, because they get no daily time off. Quite true we may remain in and take our meals, but to keep in good health we must go out and away from hospital, and in these times it is a very serious consideration. It means supporting ourselves for two months in the year.

Some hospitals have arranged to give us our coupons on days off if we apply a fortnight beforehand. Here, again, we cannot take advantage, as our time off is only arranged one week beforehand. One other thing is that the average official absolutely resents new-comers. They are spoken to as though they have no earthly right to be there, and they have got to work very hard and prove their worth before they are even tolerated by the older members of the staff. This attitude may have worked well in the old days, but it is quite unsuitable to the present. We cannot give our whole lives and thoughts to this one thing alone; all have their own difficulties to contend with. The absence of those you love and hold dear, even if they are safe so far, is in itself a constant anxiety. It is just wonderful to think what has been done and is still being done by those who are daily doing double the work formerly done. Never before was there such a shortage of staff, especially in regard to the domestic staff, and yet things go on just the same, although it is only the nurses who are doing it. These institutions cannot refuse cases to the extent that a general hospital can. We must protect the public. Most ordinary illnesses can be nursed in their own homes, but infection cannot. Trusting these few hints may help to set matters a bit straight.—Yours faithfully, M.A.B.

